# 

**Published:** 1981

**Authors:** 


JANUARY/APRIL 1981
VOLUME 96 (i/ii)
NUMBER 357/358
Published Quarterly
Annual Subscription £6 Post Free

### BRISTOL MEDICO-CHIRURGICAL JOURNAL

### Contents

3 **Editorial**

Established 1883

### BRISTOL MEDICO-CHIRURGICAL JOURNAL

Incorporating the Medical Journal of the South West

### Editorial Committee

EDITOR
*M. G. Wilson, Esq.*
*Litfield House, Clifton, Bristol, BS8 3LS*

ASSISTANT EDITOR
*Dr. G. R. Tribe*
*Southmead Hospital, Bristol, BS10 5NB*

MEMBERS
*Dr. Rhys Davies*
*Dr. M. B. Lennard*
*Dr. D. W. Wright*
*The Hon. Treasurer of the Society*
*The Hon. Secretary of the Society*

SECRETARY
*Mr. B. P. Jones, The Library, Medical School,*
*University Walk, Bristol, BS8 1TD*

### Bristol Medico-Chirurgical Society

PRESIDENT 1980/81
*Dr. N. J. Brown*

PRESIDENT ELECT
*Dr. R. P. Warin*

HONORARY SECRETARY
*Dr. H. M. Norman*
*Hillside, Vicarage Lane, Barrow Guerney*

HONORARY TREASURER
*Dr. J. Penry*
*Southmead Hospital, Bristol, BS10 5NB*

The Society was founded in 1874. In 1883 publication of the Journal began and has continued quarterly ever since then. During the years 1949 to 1962 it appeared under the title of 'The Medical Journal of the South West'.

### Notice to Contributors

*The Journal is especially concerned to provide a place for the recording of medical thought, interests, and practice in and around Bristol. It therefore welcomes articles that, as well as being of immediate interest to members, will document the local and contemporary medical scene for our successors.*

*Original articles are invited provided that they have not been published elsewhere. Illustrations should be supplied ready for reproduction. Line drawings should be in Indian ink on firm white paper or board. The author will be informed if it is found necessary to ask him to pay for the printing of photographs, etc.*

*The following contributions will be especially welcome: notes of forthcoming events, meetings held, degrees conferred, staff appointments, honours and public appointments and other local news.*

*Advice on preparation can be obtained from the Editor.*

Printed by the University of Bristol Printing Unit

### University of Bristol Faculty of Medicine

### FINAL EXAMINATION FOR THE DEGREES OF M.B., Ch.B.
**December 1980**

**PASS**
Brigid Eileen HAYDEN
Jonathan David HUCK
Michael Dennis KUCYJ
Julian Peter LOB-LEVYT
John Gareth PHILLIPS
Alexander Bruce SETON BROWNE
Robin Elizabeth TEOH
Christopher Stuart Adrian WAYTE

### FINAL EXAMINATION FOR THE DEGREE OF B.D.S. (Section II)
**December 1980**

**WITH HONOURS**
Anne Margaret BLAKEMORE
Richard Francis JOHNSON
William Philip SMITH
Rosemary Caroline THOMPSON
Andrew Philip YAXLEY

**PASS**
Stephen David ADCOCK
Richard Margrave AYRTON
Helen Patricia BARNES
Christopher Noel Alick BELL
Paula Vedora BOBISHKO
Graham Anthony BRIDGE
Dermot Brendan BROWNE
Gillian Margaret Anne CLARK
Rebecca Clare CRAVEN
Ian Arthur CROUCH
Rosamund Estelle CROWTHER
John Victor DAVEY
Mark Andrew DE COTHI
Robin Donald Blundell DICKSON
Helen Elizabeth DOUGLAS
Judith Margaret EDWARDS
Karen Margaret ELLEY
Stephen Drummond FAIRLEY
Michael Joseph FRAIN
Peter Esmond FROUD
Warren GAMBLIN
Howard Robert GEORGE
Jean Elizabeth Anne GULLEY
Jonathan Paul HAYTER
Jonathan Paul HINDLE
Peter Robert JONES
Stephen Douglas JONES
Denzil Michael John LE ROY
David George LOUGHTON
Terence William MASON
Rajesh MAYOR
Gary Edward MENDHAM
Hiten Ramanbhai PATEL
Karen Charlotte PIERCE
Ivan Keith POINTON
David Stanley RABY
Gillian RATCLIFF
Lindsay Howard RICH
Ryszard Stanislaw SAMLIK
Jillian Patricia SMITH
Richard Peter TONKS
Andrew Charles TOY
Gillian WORTHY

### FINAL EXAMINATION FOR THE DEGREE OF B.D.S (Section I)
**December 1980**

**PASS**
Christopher David ACKLAND
Stephen Richard ADAMS
Guy John ATHERTON
Diana Louise BARRETT
Noorali Abdulali BHATIA
Janice Elizabeth BOSWELL
Brant Nigel CHAPMAN
Neil Jonathan CHAPMAN
Henry Charles CLOVER
Graham Richard COCKROFT
Richard Simon DAVIES
Richard Francis DRAGE
Mark William DRAPER
Stephen Alan FAYLE
Geraldine Victoria FELLOWS
Penelope Susan GADD
Karen Jane GLOVER
Ian Phillip HARDY
Kenneth Wade HEMMINGS
Simon Charles HIPWELL
Kai Mun HUEN
Susan Linley HUMPHERSON
Peter Nils HUNTLEY
Jonathan Cunliffe HYDE
Richard Llewelyn Graham JONES
Jennifer Grace KERSHAW
Mehmet MANISALI
Andrew MARSHALL
Timothy Andrew MORRIS

Timothy Arthur Robert PEACOCK
Steven John PREDDY
David Edward ROBERTS
Stuart Richard SMITH
Caroline Ann SOUTHALL
Alison Wendy STRONG
Heather Anne TOTTEN
Janette Ann WALKER
Alison Clare WILLIAMS
Stephen John WOOD
Clare Elizabeth WOODHOUSE

**THE DEGREE OF DOCTOR OF MEDICINE**
**January 1981**
**Dissertations Approved**

Timothy Joseph DAVID
Ian Zem MacKENZIE
Adrian Philip MANNING

**THE DEGREE OF DOCTOR OF PHILOSOPHY**
**January 1981**
**Dissertations Approved**

Brian Geoffrey Hugh LEVERS
Christine Anne MIDWINTER
Maria Guadalupe ORTEGA PIERRES
Akram Majid RASHID
Alastair James Scott SUMMERLEE
Leonidas VASILIADIS

**THE DEGREE OF MASTER OF SCIENCE IN MEDICAL SCIENCES**
**January 1981**
**Dissertation Approved**

Abdulmonam Mabrouk AL-JIBANI

**THE DEGREE OF DOCTOR OF MEDICINE**
**March 1981**
**Dissertations Approved**

John Tamblyn BOURNE
Janet Dorothy MILNE

**THE DEGREE OF DOCTOR OF PHILOSOPHY**
**March 1981**
**Dissertation Approved**

Askary RADFAR

**THE DEGREE OF MASTER OF SCIENCE IN MEDICAL SCIENCES**
**March 1981**
**Dissertation Approved**

Peter Michael WEST

